# Smart Soil Water Sensor with Soil Impedance Detected via Edge Electromagnetic Field Induction

**DOI:** 10.3390/mi13091427

**Published:** 2022-08-29

**Authors:** Hao Tian, Chao Gao, Xin Zhang, Chongchong Yu, Tao Xie

**Affiliations:** 1 School of Artificial Intelligence, Beijing Technology and Business University, Beijing 100048, China; 2 China Light Industry Key Laboratory of Industrial Internet and Big Data, Beijing Technology and Business University, Beijing 102448, China

**Keywords:** soil water sensor, edge electromagnetic field induction, normalized calibration method, performance analysis

## Abstract

To address the problems in the calibration of soil water content sensors, in this study, we designed a low-cost edge electromagnetic field induction (EEMFI) sensor for soil water content measurement and proposed a normalized calibration method to eliminate the errors caused by the measurement sensor’s characteristics and improve the probe’s consistency, replaceability, and calibration efficiency. The model calibration curve-fitting coefficients of the EEMFI sensors were above 0.98, which indicated a significant correlation. The experimental results of the static and dynamic characteristics showed that the measurement range of the sensor varied from 0% to 100% saturation, measurement accuracy was within ±2%, the maximum value of the extreme difference of the stability test was 1.09%, the resolution was 0.05%, the delay time was 3.9 s, and the effective measurement diameter of the EEMFI sensor probe was 10 cm. The linear fit coefficient of determination of the results was greater than 0.99, and the maximum absolute error of the measurement results with the drying method was less than ±2%, which meets the requirements of soil water content measurement in agriculture and forestry fields. The field experiment results further showed that the EEMFI sensor can accurately respond to changes in soil water content, indicating that the EEMFI sensor is reliable.

## 1. Introduction

Soil water content is an important environmental parameter in agriculture and, therefore, plays a critical role in the plant growth process [1,2]. Green et al. reported that soil water content greatly affects the long-term uptake of soil nutrients by crops [3]; therefore, the accurate measurement of soil water content is essential to the study of the relationship between soil moisture and the ecological mechanism of maintaining plant growth and improving the precision of crop irrigation [4,5,6]. The main methods for quickly measuring soil water content are the drying, radiometric, and nuclear magnetic resonance methods [7,8,9]. The drying method is currently the most standard internationally recognized measurement method; however, its time-consuming and non-repeatable characteristics make it difficult to apply to online real-time measurements [2]. To achieve rapid measurement of soil water content, tensiometers based on the water suction of the soil were used in early agricultural production [10,11]. However, the relationship between soil water absorption and soil water content measured by the tensiometer is not linear, resulting in a large measurement error. Subsequently, the neutron attenuation-based soil water content measurement technique has also been applied for the rapid measurement of soil water content [12]; however, personnel using neutron water meters must be professionally trained to prevent radiation exposure from neutron sources, and the equipment is expensive. The nuclear magnetic resonance (NMR) method detects water content by detecting the proportional relationship between the initial amplitude of the NMR signal and the amount of free water in the soil sample to be measured, which has high precision and accuracy [13,14]. However, the NMR method requires large instruments and expensive equipment, which is unsuitable for portable measurements.

In recent years, owing to the development of spectroscopic technologies, new detection methods based on near-infrared spectroscopy and electromagnetic waves have emerged. Near-infrared soil water content measurement is a noncontact, nondestructive measurement method with the advantages of a short detection time, being harmless to the human body, and having high sensitivity, which has gained the attention of many researchers [15,16]; however, the current equipment is costly and is mostly used under laboratory conditions. The electromagnetic wave method is the most widely used soil water content measurement technique and is mainly divided into time-domain reflection (TDR), time-domain transmissometry (TDT), frequency-domain reflection (FDR), and standing wave rate (SWR) methods. Feldegg (1969) was the first to use TDR for the study of the electrical properties of liquids [17]. Topp et al. proposed the use of TDR in the field of soil water content measurement, and their proposed Topp equation led to a new period of soil water content detection, which has continued to receive attention from a wide range of scholars and in the development of related water measurement devices [18,19]. The TDR method has the advantage of high measurement accuracy and does not require calibration but is more costly [20,21,22]. Meanwhile, soil water content measurement technology based on TDT and FDR has been developed along with related products [23,24]. Miller et al. proposed measuring soil water content based on SWR [25], and Yiming et al. carried out further related studies and developed an SWR sensor [26,27,28]. The SWR technique for measuring soil water content has the advantage of low cost but requires calibration for each soil type before it can be used.

In order to solve the disadvantages of the time-consuming and laborious calibration process of soil moisture sensors, and to reduce the cost of soil moisture sensors to make them more convenient for widespread use, in this study, we designed an edge electromagnetic field induction (EEMFI) sensor for soil water content measurement based on the method of impedance detection and proposed a normalized calibration method to eliminate the errors caused by the measurement circuit itself and improve the consistency of the output. The EEMFI sensor designed in this study is inexpensive, with a cost of USD 100, and provides an effective and low-cost method for the rapid measurement of soil water content in agriculture, forestry, and ecological environments. The main research contents of this paper are as follows: (1) developing the EEMFI sensor based on the method of impedance detection, (2) proposing a normalized calibration method, and (3) analyzing the measurement performance of the EEMFI sensor to verify the accuracy and reliability of the measurements.

## 2. Materials and Methods

### 2.1. Experimental Materials

**Figure 1:**
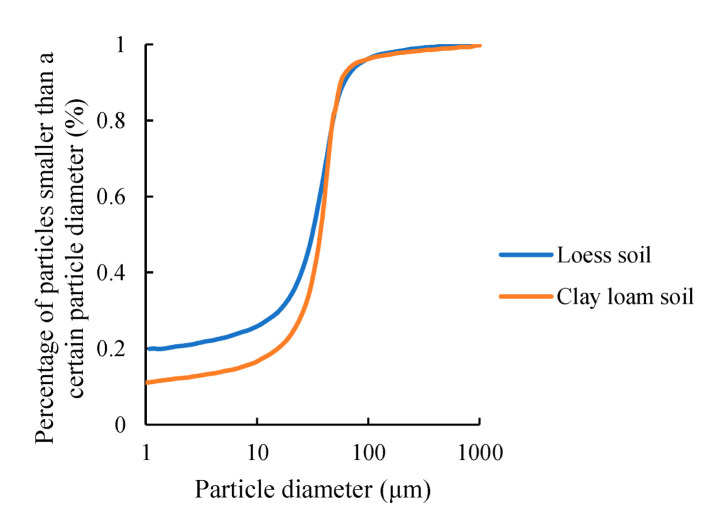
The grain size distribution curves of the soils.

The soil samples were clay loam (11% sand mass fraction, 71% powder mass fraction, 18% clay mass fraction) collected from the Bajia experimental site in Haidian District, Beijing (116.34° E, 40.00° N) and loess (15% sand mass fraction, 65% powder mass fraction, 20% clay mass fraction) collected from an apple orchard in Zhen Yuan County, Qingyang City, Gansu Province (107.03° E, 35.54° N). The grain size distribution curves of the soils are shown in Figure 1.

The field measurement site was an apple orchard in Zhenyuan County, Qingyang City, Gansu Province, where an unplanted plot was randomly selected and a rainfall sensor (PG-210/YL-CG, Tianyuhuanke, Beijing, China) and two EEMFI sensors were installed: Sensor 1 at 5 cm from the soil surface and Sensor 2 at 15 cm from the soil surface. Data were collected and saved every 10 min using a data collector (M10001, Smacq, Beijing, China) for the experimental period from 10 July 2020 to 10 August 2020.

### 2.2. EEMFI Sensor Principle

**Figure 2:**
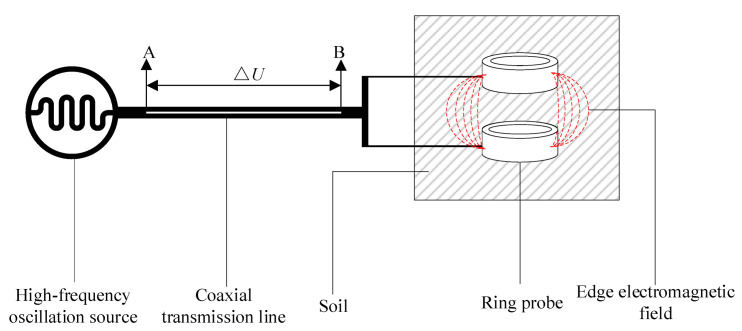
EEMFI sensor measurement schematic.

When the high-frequency electromagnetic wave generated by the high-frequency signal source reaches the probe along the coaxial transmission line, the electromagnetic wave is reflected back, owing to the impedance mismatch between the probe and transmission line, and the incident and reflected waves form a potential difference at both ends of the coaxial transmission line [25,29,30], as shown in Figure 2. During the transmission process, the bimetallic ring probe is positioned within the soil, and any impedance change causes a change in the magnetic field medium, which reflects back along the electromagnetic wave. This leads to the ends of the coaxial transmission line having different potentials, and this change in voltage represents the impedance change in the probe in the soil. As the water content is the main factor causing a change in impedance, the change in voltage between the ends of the transmission line can be used to calculate the soil water content.

The high-frequency signal source used was a 100 MHz sine wave, and the characteristic impedance of the coaxial transmission line was 50 Ω. The soil dielectric constant at this frequency can be expressed by the following formula [31]:

(1) $$\varepsilon =\varepsilon^{\prime }-j\left(\varepsilon^{\prime \prime }+\frac{K}{2\pi f\varepsilon_{0}}\right),\;$$

where ε is the complex dielectric constant of the soil; $\varepsilon^{\prime }\;\mathrm{and}\;\varepsilon^{\prime \prime }$ are the real and imaginary parts of *ε*, respectively; j denotes the imaginary part of a complex number (purely mathematical notation with no real physical meaning); *K* is the conductivity of the soil to be measured; *f* is the frequency of the excitation signal; and $\varepsilon_{0}$ is the dielectric constant in air. The impedance of the probe determines $\varepsilon^{\prime }$, the dielectric loss determines $\varepsilon^{\prime \prime }$, and the coaxial transmission line impedance can be calculated using Equation (2).

(2) $$Z=\frac{60}{\sqrt{\varepsilon }}ln\left(\frac{R_{a}}{R_{b}}\right),$$

where $R_{a}$ is the radius of the sensor probe, and $R_{b}$ is the radius of the circle formed outside the sensor probe. The reflection coefficient ρ of the transmission line is calculated using Equation (3).

(3) $$\rho =\frac{Z_{P}-Z_{L}}{Z_{P}+Z_{L}},$$

where $Z_{P}$ is the impedance at the sensor probe, and $Z_{L}$ is the impedance of the coaxial transmission line. The coaxial transmission line excitation signal is $U_{a}$ given by Equation (4).

(4) $$U_{a}=E\sin\left(2\pi ft\right)$$

where *E* is the amplitude of the excitation signal, and *t* is the excitation signal transmission time. The excitation signal at the output of the coaxial transmission line is

(5) $$U_{b}=E\sin\left(2\pi ft\right)+E\rho \sin\left(2\pi f\left(t-\frac{2L}{v_{s}}\right)\right),\;$$

where *L* is the distance between the ends of the coaxial transmission line, and $v_{s}$ is the transmission speed of the excitation signal on the coaxial transmission line. When the length of the transmission line is 1/4 of the wavelength, that is, $L=v_{s}/4f,$ then

(6) $$U_{b}=E\left(1-\rho \right)\sin\left(2\pi ft\right).$$

When sin(2πft)=1, the value of the excitation signal at its peak is

(7) $$U_{b}=E\left(1+\rho \right),\;$$

and the excitation signal voltage at the other end of the coaxial transmission line is

(8) $$U_{a}=E\left(1-\rho \right).\;$$

Therefore, the voltage difference between the two ends of the transmission line is

(9) $$∆U=U_{b}-U_{a}=2E\rho =2E\frac{Z_{P}-Z_{L}}{Z_{P}+Z_{L}}.\;$$

According to Equation (9), the change in soil water content causes the voltage at both ends of the coaxial transmission line to change, and the voltage difference is linearly proportional to the water content after adjustment through the circuit; thus, the water content of the soil can be obtained by measuring the voltage difference at both ends of the transmission line after calibration.

### 2.3. EEMFI Sensor Hardware System

**Figure 3:**
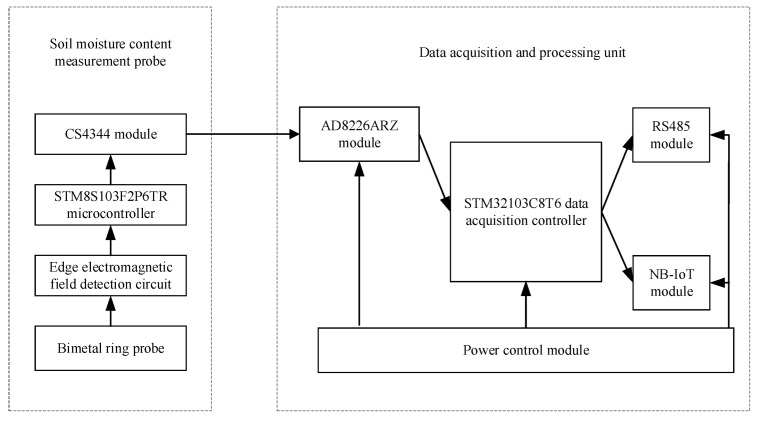
Hardware circuit system block diagram.

**Figure 4:**
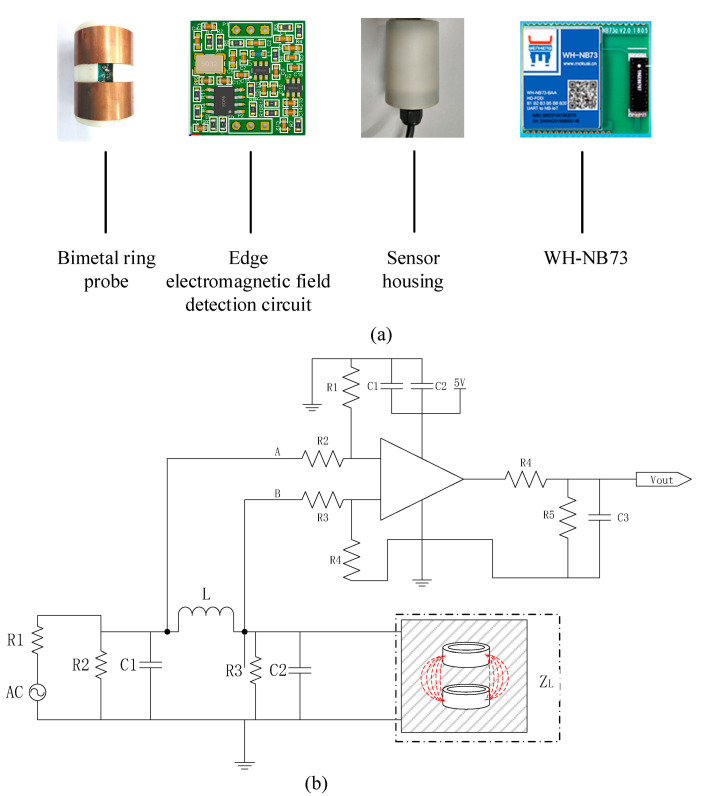
Photographs and detection circuit of EEMFI sensor: (**a**) photographs of EEMFI sensor and (**b**) schematic diagram of edge electromagnetic field detection circuit.

The hardware circuit mainly included the soil water content measurement probe and a data acquisition and processing unit. The hardware circuit system block diagram is shown in Figure 3. The data acquisition processing unit included a data acquisition controller (STM32103C8T6, STMicroelectronics, Geneva, Switzerland), an analog-to-digital converter (AD8226ARZ, Analog Devices Inc., Wilmington, MA, USA), a power control module (K7805-1000R3, Dexu Electronics, Shenzhen, China), a clock control module (RX-8025T, Epson Toyocom, Nagano, Japan), an RS485 module (MAX485ESA, Milkin, Shenzhen, China), and an NB-IoT module (WH-NB73, Wenheng, Jinan, China). The soil water content measurement probe was developed and designed by us, which included a bimetal ring probe (material is brass, external diameter is 5 cm, edge thickness is 1.5 mm, and height is 3 cm), an edge electromagnetic field detection circuit (Figure 4b), a microcontroller (STM8S103F2P6TR, STMicroelectronics, Geneva, Switzerland), and a digital-to-analog converter (CS4344, Cirrus Logic, Austin, TX, USA). The EEMFI sensor operating voltage was 5 V. The water content measurement probe used the STM8S103F2P6TR processor to collect the edge electromagnetic field detection circuit output voltage signal by writing the normalized calibration parameters and calculating the corresponding digital signal. To collect the water content output voltage value, the CS4344 converter output the voltage signal to the main board through the AD8226ARZ converter after data processing. The measurement results are also uploaded to the Internet of Things (IoT) cloud platform (http://cloud.usr.cn, accessed on 15 January 2021) in real time through the NB-IoT module. The data were communicated according to the recommendation standard 485 (RS485) protocol. A physical diagram of the sensor is shown in Figure 4a, and the cost of the EEMFI sensor was only USD 100 (Table 1); the prices of some commonly used soil moisture sensors are shown in Table 2. The bimetal ring probe was processed by contacting a local metal fabricator for the hardware measurement circuit. We designed the circuit ourselves and commissioned a PCB fabrication company to make the measurement circuit board through the Taobao website, and the housing was independently printed using 3D printing equipment.

### 2.4. Calibration of EEMFI Sensors Based on Normalization Method

Sensor calibration is essential to sensor design, manufacture, and use [32,33]. All sensors must be calibrated after manufacturing and assembly to ensure measurement accuracy. Soil water content sensors often have large measurement errors, owing to the variability in the sensor characteristics, different types of soils being measured, and improper use. Therefore, we proposed a normalized calibration method to eliminate the errors caused by the characteristics of the EEMFI sensor by normalizing the mapping relationship between the output voltage of the standing wave circuit and the standard dielectric constant. Subsequently, the mapping relationship between the dielectric constant and water content was established for different soils to calibrate the sensor twice to improve the accuracy and convenience of the EEMFI sensor.

Normalized calibration samples were used to calibrate the EEMFI sensor probes with different dielectric constant solutions to establish a relationship model between the output voltage value of the edge electromagnetic field detection circuit and the dielectric constant to eliminate errors in the measurement of the dielectric constant caused by the different characteristics of the EEMFI sensor devices. To facilitate calibration and operator safety, nontoxic anhydrous ethanol (C_2_H_6_O, dielectric constant 24.5) and acetic acid (CH_3_COOH, dielectric constant 6.15) were used to configure solutions with different dielectric constants in different volume ratios with water (H_2_O, dielectric constant of 80) to a total volume of 250 mL. The dielectric constants of these solutions are listed in Table 3.

**Figure 5:**
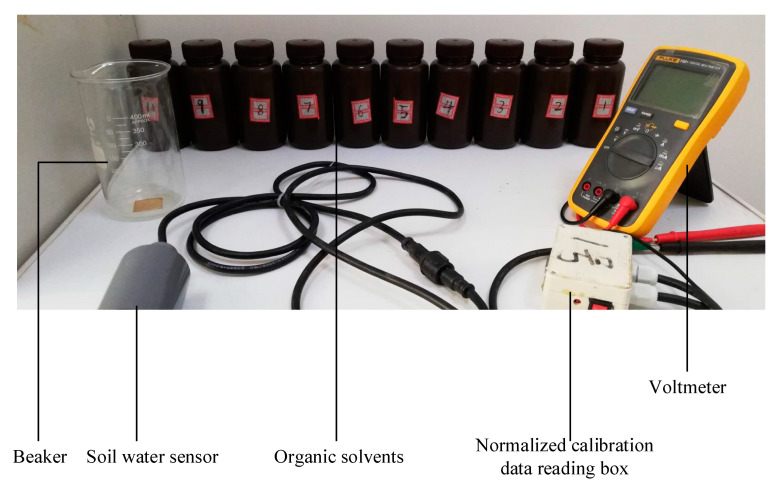
Photograph of normalized calibration.

The configured solutions with different dielectric constants were poured into the beaker in turn; then, the EEMFI sensor probe was placed into the beaker and the normalized calibration data reading box, with one port for connecting to the sensor and the other port for connecting to the voltmeter. The voltage value output from the sensor probe was then recorded with a voltmeter. The normalized calibration site is shown in Figure 5. The above steps were repeated to measure the corresponding voltage values and dielectric constant solutions and to perform data fitting to establish the normalized calibration model in Equation (10).

(10) $$\varepsilon_{l}=a∆U^{3}+b∆U^{2}+c∆U+d\;$$

where $\varepsilon_{l}$ is the organic solvent dielectric constant; ∆U is the standing wave circuit output voltage value; and a, b, c, and d are the constant term coefficients. The EEMFI sensor probe measurement of the dielectric constant was in the range of 1–80, to comply with the actual production and device’s life cycle needs. Due to the inclusion of the STM8S103F2P6TR microcontroller and CS4344 digital-to-analog converter (see Section 2.3), the EEMFI water content measurement probe can output a 0–2.5 V analog signal. The model for the conversion of the measured dielectric constant to the corresponding voltage using the digital-to-analog converter is as follows:

(11) $$U=0.0316\varepsilon_{l}-0.0316,\;$$

where U is the analog voltage output by the digital-to-analog converter, which yields the values of coefficients a, b, c, and d. By writing each value into the memory of the STM8S103F2P6TR microcontroller, the measured dielectric constant can be converted to an analog signal of 0–2.5 V for output. By establishing a normalized calibration model and fitting the relationship between the output voltages of the edge electromagnetic field detection circuit and the standard dielectric constant solution, the variability in the EEMFI sensor and measurement errors caused by manufacturing and processing errors can be eliminated, the consistency of the EEMFI sensor probe output can be improved, and the measured dielectric constant can be converted into an analog voltage output. The analog voltage output improves the replaceability and versatility of the EEMFI sensor probe and is easy to use.

Owing to the variability in various soils, further secondary calibrations based on different soils were carried out to make the measurements highly accurate. Experimental soil samples (5 kg) were dried in a laboratory drying oven (105 °C, 48 h) and individually sieved through a 40-mesh sieve. Water was added to the sample and stirred for 10 min until the water and soil were fully mixed, and the water content of the soil was uniform. Each sample was then divided into eight equal parts, and the first subsample was added to the PVC barrel (diameter 40 cm, height 25 cm) and compacted evenly with a nylon rod (diameter 50 mm, length 50 cm). The eight subsamples were added to the calibration barrel in turn; the barrel was sealed and left for 48 h to ensure the water transport in the calibration barrel reached equilibrium. The EEMFI sensor was buried in the soil sample calibration bucket, and the water content measurement probe output voltage (output by the digital-to-analog converter) was recorded. The samples in the calibration bucket were sampled with a ring knife (100 mL), and two of these subsamples were taken and dried in a drying oven (105 °C) for 24 h. The water content was calculated using the drying method, and the water content of the two drying samples was averaged to obtain the water content of the current soil sample. By adding different volumes of water to the samples to obtain different amounts of water content in the experimental samples, each soil sample was configured with eight different amounts of water content. The voltage measured by the sensor was recorded against the corresponding water content while the sensor was placed in the air (the water content was assumed to be 0), and the output voltage value of the EEMFI sensor was recorded. A linear fit of the voltage and water content was performed to establish the equation for the quadratic calibration as follows:

(12) θ=KU+B, 

where θ is the water content of the soil, U is the analog voltage output from the digital-to-analog converter, and K and B are the calibration coefficients determined by the soil quality. The calibration coefficients K and B can be saved in the data acquisition and processing unit of the EEMFI sensor in advance so that the calibration coefficients can be called at any time when measuring different soils.

### 2.5. Performance Analysis of EEMFI Sensors

The sensor’s static characteristics represent the characteristics of the relationship between the input and output of the sensor when the input signal is either constant or changing very slowly. According to the requirements of the water content sensor, in this study, we analyzed the measurement range, measurement accuracy, stability, and resolution [34,35] of the sensor.

The dynamic characteristics of the sensor represent the sensor’s response to the input quantity that changes with time. The EEMFI sensor in the air or soil in this process is the input signal, and the input signal at the time of the first-order step signal can be obtained from the dynamic characteristics of the sensor [36,37].

### 2.6. Sensitive Areas for Water Measurement with EEMFI Sensor

The sensitive area of a sensor represents the range of soil that can be effectively sensed during the measurement process and is an important indicator of sensor performance. In this study, we used a high-frequency electromagnetic simulation software program (HFSS v 13.0, Ansys, Pittsburgh, PA, USA) to simulate the probe model. The frequency of the excitation signal was set to 100 MHz, and the media boundary of the probe was a cube with side lengths of 30 cm. The cube was larger than the diameter of the ring-type probe to simulate the probe in open earth and analyze the shape and range of the water content measurement area by simulating the electromagnetic field of the probe.

### 2.7. Performance Verification of EEMFI Sensor

To verify the actual performance of the EEMFI sensor designed in this study, the accuracy of the measurement results was verified using a TDR water content sensor (TRIME-PICO, IMKO, Germany, measurement accuracy ±2%, measurement range 0–100%) and the drying method. Loess soil and clay loam soil samples for measurement were configured in the laboratory, and the measurement sample of each soil was configured with eight gradients of water content. The EEMFI and TDR sensors were used to measure the soil water content of the samples. In addition, seven sites were randomly selected from experimental sites in Haidian District, Beijing, and the water content of the soil at each site was measured using the EEMFI sensor. The samples were simultaneously dried at the corresponding sites using a ring knife, and the corresponding water content of the soil was calculated using the drying method.

## 3. Results and discussion

### 3.1. Calibration of EEMFI Sensors

**Figure 6:**
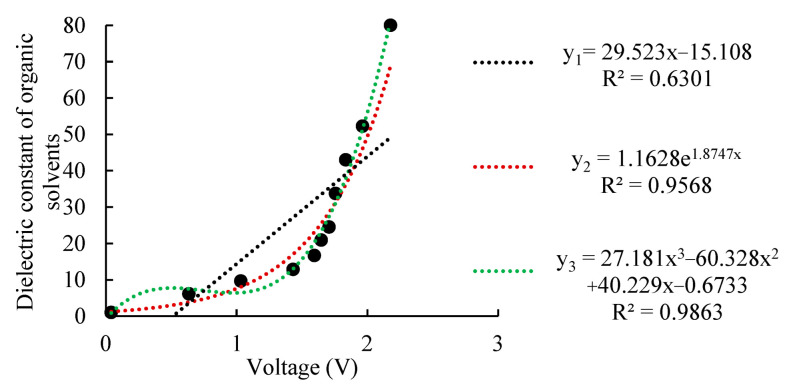
Data fit under normalized calibration.

Using the techniques described in Section 2 [32,33,38], the output voltage of the edge electromagnetic field detection circuit under the corresponding dielectric constant solution was measured, and linear, exponential, and third-order polynomial fits were selected. Figure 6 shows the fitted coefficients of determination $R^{2}$ for the three forms of fit, which were 0.6301, 0.9568, and 0.9863, respectively. The best fit was clearly obtained using the polynomial fit; therefore, the constant term coefficients of the normalized calibration model in Equation (10) were a=27.181, b=−60.328, c=40.229, and d=−0.6733. By writing the constant term coefficients a, b, c, and d into the memory of the probe’s STM8S103F2P6TR processor, the normalized calibration of the EEMFI sensor probe was completed. Normalizing the calibration so that every probe outputs a 0–2.5 V analog signal ensures the easy interchangeability of any two probes, thus improving the replaceability and versatility of the EEMFI sensor probe.

Subsequently, a secondary calibration was performed for different types of soil. The fitting results of the output voltage value of the water content measurement probe and the water content of the soil are shown in Table 4. The voltage and soil water content had a good linear relationship, and the primary linear fitting coefficient of determination $R^{2}$ exceeded 0.98. The values of K and B were used as calibration coefficients for different soil types, and their values were written into the data acquisition processing unit and memory of the STM32103C8T6 data acquisition controller.

### 3.2. Advantages of EEMFI Sensor Calibration Based on Normalization Method

**Figure 7:**
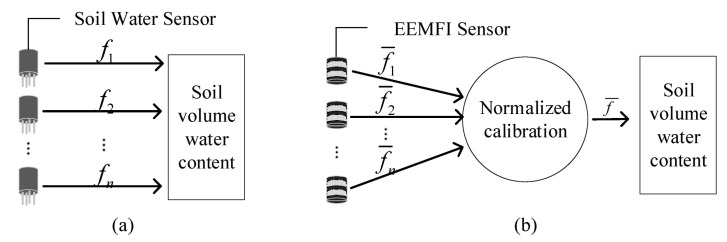
Comparison of calibration methods: (**a**) traditional calibration method and (**b**) normalized calibration method.

Figure 7a shows the calibration of a conventional soil water content sensor by establishing the mapping relationship between the sensor and water content ($f_{1}$,$f_{2},\ldots$,$f_{n}$) under different soil conditions. Xu et al. pointed out that each sensor corresponds to its own mapping relationship due to the characteristics of the sensor and the soil texture [28]; therefore, once the soil type or sensor changes, the sensor needs to be recalibrated. *n* sensors are used in *m* soil types, and a total of *nm* calibrations are needed [27]. If a sensor is damaged, the replacement sensor needs to be recalibrated.

Figure 7b shows the normalization calibration schematic by normalizing the mapping relationship between the output voltage and the dielectric constant of the EEMFI sensor (${\bar{f}}_{1}$,$\bar{f_{2}}$···${\bar{f}}_{n}$) and eliminating the influence of the EEMFI sensor device. Subsequently, the mapping relationship between the dielectric constant and water content $\bar{f}$ is established for different soils, with *n* sensors in *m* soils. For use with *m* soils, the EEMFI sensor is first calibrated *n* times to make its output consistent, and then only *m* secondary calibrations are required for the soil, and a total of *n* + *m* calibrations are needed. Therefore, we called the number of calibrations required g. The number of sensor calibrations is $g^{\prime }$ under the conventional method and $g^{\prime \prime }$ under the normalized method. $g^{\prime }$ and $g^{\prime \prime }$ can be calculated using Equations (13) and (14).

(13) $$g^{\prime }=n\cdot m\;$$

and

(14) $$g^{\prime \prime }=n+m\;$$

**Figure 8:**
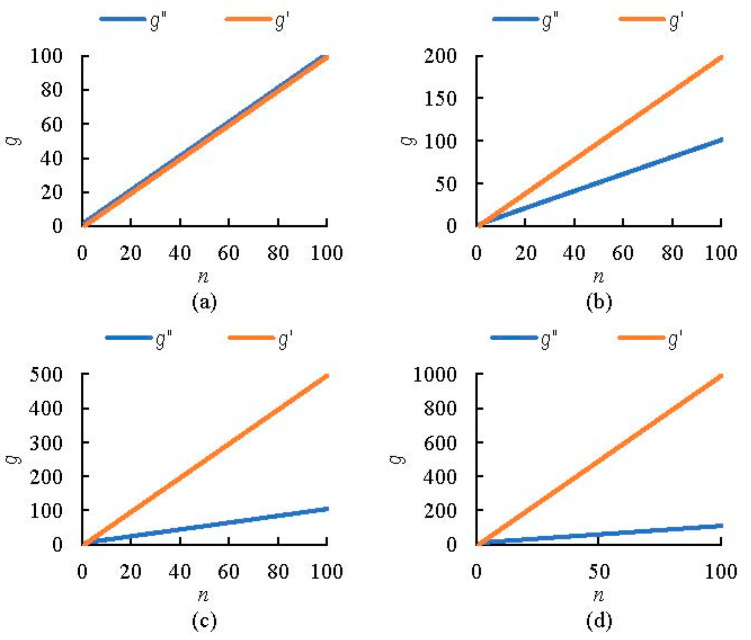
Comparison of the number of calibrations: (**a**) *m* =1, (**b**) *m* = 2, (**c**) *m* = 5, and (**d**) *m* = 10.

The results of the number of calibrations required for the two calibration methods are shown in Figure 8. When there was only one soil texture (*m* = 1), the number of calibrations was essentially the same for both sensors. When the number of soil types increased, the number of calibrations required for the sensor under the traditional method became significantly greater than the number of calibrations for the sensor under the normalized method; the greater the number of soil textures, the greater the difference between the two. Rowlandson et al. pointed out that the traditional calibration method can be a good solution for errors caused by differences between soil texture and sensors [33], but it is very labor-intensive [39], and the results in Figure 8 also support this conclusion. In contrast, our proposed sensor calibration based on the normalization method effectively reduces the calibration workload and improves the replaceability of the sensors, which is more convenient for practical use.

### 3.3. Analysis of Static and Dynamic Characteristics of EEMFI Sensors

The measurement range indicates the maximum and minimum values measured by the sensor [34,35]. Figure 6 shows that the dielectric constant range measured by the EEMFI sensor probe was 1 to 80; therefore, the theoretical measurement range of the EEMFI sensor was 0% to 100% (the amounts of water content for the air and pure water are assumed to be 0% and 100%, respectively). However, in practice, 100% water content in the soil sample is difficult to configure, and the maximum measurement range of the soil water content sensor is usually expressed in terms of the saturated water content internally; therefore, the actual measurement range of the EEMFI sensor was 0% to saturation.

Sensor measurement accuracy indicates the reliability of the measurement results and is generally expressed as a percentage of the ratio of the maximum error to the full range [40]. For clay loam and loess soils, three samples of each soil were configured with different amounts of water content, and their water content values were measured by the EEMFI sensor. In total, 10 measurements were taken for each sample, and the maximum error value was used to calculate the measurement accuracy. The full-scale range was selected according to the requirements of the measurement accuracy calculations of commercial water content sensors, and the water content of the soil samples was obtained by the drying method. The measurement accuracy data are shown in Table 5. The maximum accuracy of the EEMFI sensor was 1.98% and within 2% for the three soils. Studies have shown that soil moisture sensors can be used in agroforestry applications when the accuracy of the measurement is within 2% [39,41]; thus, our experimental results also prove that the EEMFI sensor meets the practical requirements for soil moisture measurement in agriculture and forestry.

**Figure 9:**
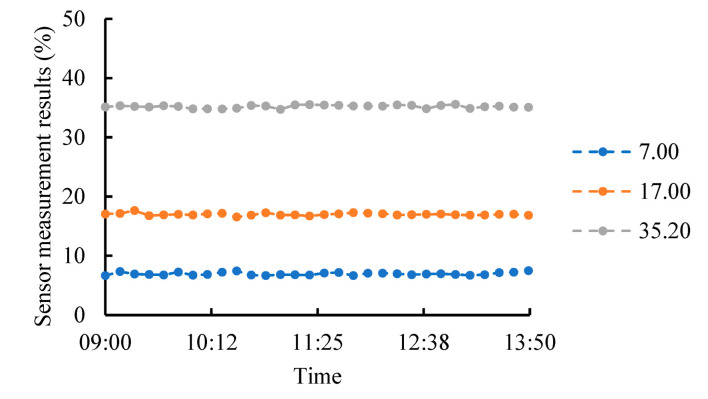
EEMFI sensor measurement stability analysis.

Stability indicates the variability of the sensor output over a long period in the same environment or under multiple measurements [40,42]. The EEMFI sensor was placed into configured soil samples (sample volume water content of 7.00%, 17.00%, and 35.20%), and the sensor measurements were read at ten-minute intervals, with 30 measurements per sample. The data variation curve obtained is shown in Figure 9. The maximum standard deviation of the measurement results was 0.24%, and the maximum extreme value distribution was 1.09%. The fluctuation in the sensor measurement results was very small, and from the results, it was obvious that the sensor output results had good stability [40,42] and could be used for repeated measurements.

Resolution indicates the ability of the sensor to sense the smallest change in the measured occurrence [4,40]. The EEMFI sensor designed in this study detects changes in the soil dielectric constant caused by a change in soil water content through the water content measurement probe and samples the output voltage through the AD8226ARZ analog-to-digital converter. The EEMFI sensor resolution is determined by the sampling accuracy of AD8266ARZ, which is 0.8 mV, corresponding to a resolution of 0.05%.

**Figure 10:**
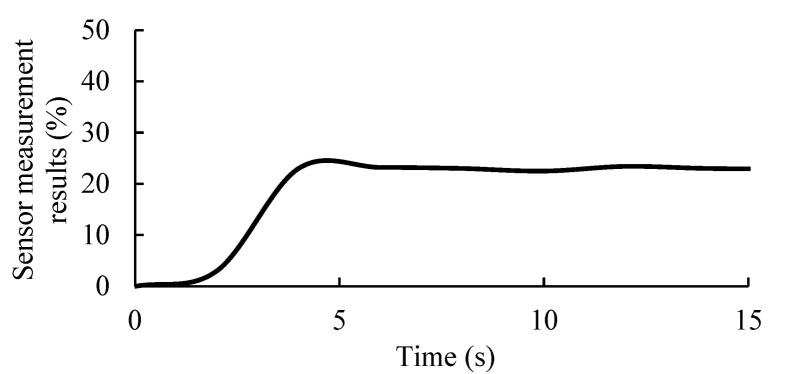
EEMFI sensor dynamic characteristic curve.

The dynamic characteristic curve of the EEMFI sensor was plotted according to the recorded data (see Figure 10), and the relevant dynamic characteristic indices were calculated (Table 6). The experimental results showed that the sensor delay time was 3.9 s, and for agriculture and forestry requirements, a delay of 3.9 s does not affect the use of the sensor [43].

### 3.4. Analysis of Sensitive Areas for Soil Water Content Measurement

**Figure 11:**
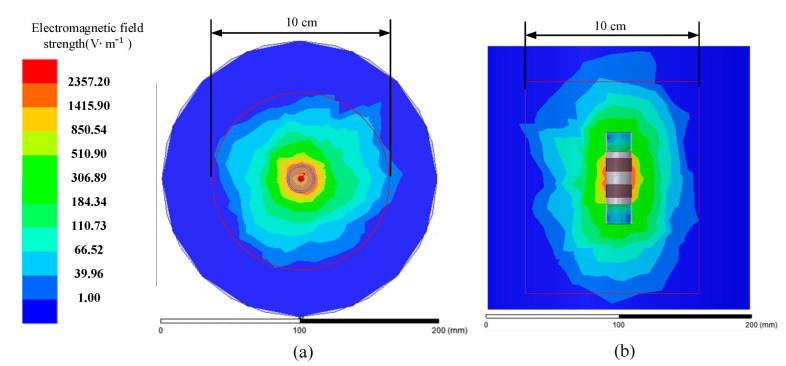
Electric field distribution of the bimetallic ring probe: (**a**) top view and (**b**) side view.

Kafarski et al. analyzed soil moisture probes via electromagnetic simulation and showed that the electromagnetic radiation range of the ring probe is closely related to the range of moisture variation it can measure [44]. The simulation results (Figure 11) using the HFSS software showed that the closer to the outer edge of the probe, the stronger the electromagnetic field, and vice versa. Clearly, the simulation results were consistent with Kafarski’s conclusions. When the electromagnetic field was more than 10 cm in diameter, the electromagnetic strength was less than 1 V/m. At this time, the EEMFI sensor probe was unable to effectively sense changes in the surrounding magnetic field, which indicated that the EEMFI sensor’s sensitive range for the probe was 0–5 cm from the center, or a measurement diameter of 10 cm.

### 3.5. Comparative Verification of Water Measurement Performance of EEMFI Sensors

**Figure 12:**
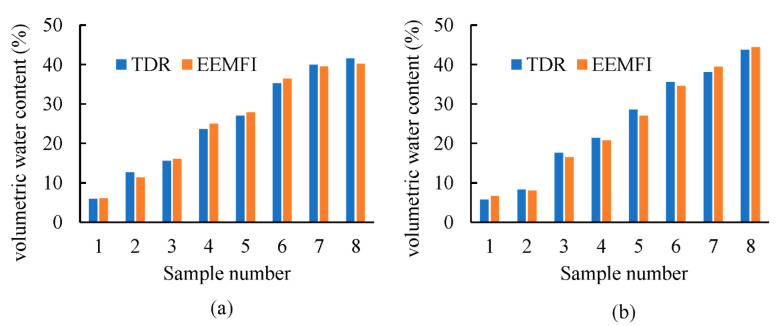
Comparison of accuracy of sensor measurement results: (**a**) clay loam soil and (**b**) loess soil.

The soil water contents of the samples to be measured were determined under laboratory conditions with the EEMFI and TDR sensors, and a comparison of the measurement results is shown in Figure 12. In the clay loam and loess samples, the soil water content measured by both sensors was nearly the same. A linear fit analysis was performed for the EEMFI and TDR sensor measurement results and the fitted coefficients of determination were 0.9932 and 0.9937, respectively. Both fitted coefficients of determination were greater than 0.99, which proved that the EEMFI and TDR sensor measurement results had a good linear correlation, indicating that the accuracy of the EEMFI sensor results are comparable to those of the TDR sensor [34,45,46] and meet the practical application requirements. However, compared with the TDR sensor, the EEMFI sensor designed in this study does not have the disadvantages of easy bending of the probe during use and high cost.

The amounts of the soil water content of the samples from the eight experimental sites in Haidian District, Beijing, obtained by using the EEMFI sensor and drying method are shown in Table 7. The results obtained by using the EEMFI sensor and drying method were nearly consistent, with a maximum absolute error of 1.74%, indicating that the practical requirements of a maximum absolute error < ±2% for soil water content measurement are met [34,45,46,47].

### 3.6. Field Experiments

**Figure 13:**
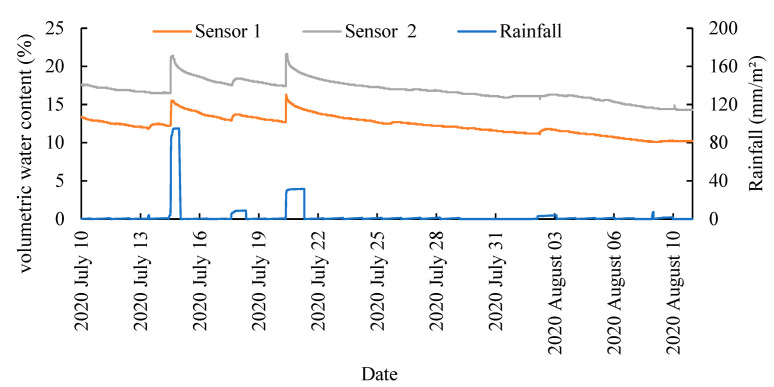
Field measurement results.

The results of the field experiments are shown in Figure 13. When rainfall increased, the measured water content of the soil also increased, which proved that the sensor can accurately respond to changes in the soil water content. The water content of the soil measured by Sensor 2 was significantly greater than that of Sensor 1, which proved that the closer to the soil’s surface, the stronger the evaporation and infiltration of water, and the weaker the soil’s ability to retain water [48,49,50]. Additionally, the rapid increase in soil water content after rainfall not only indicated a significant relationship between rainfall and soil water content but also indicated that rainfall is the main source of water in the soil [51,52]. However, controlling soil irrigation by hand is the most important tool in plant cultivation [53,54].

### 3.7. Advantages and Limitations of EEMFI Sensors

The EEMFI sensor designed in this study was calibrated using the normalization method, which greatly reduced the calibration workload of the soil water content sensor and improved the replaceability of the sensor during use. Moreover, the cost of the EEMFI sensor was only 100 USD, which is cheap, and it will be easy to use in large quantities in agriculture and forestry involving soil water content detection. However, due to the inconvenience of travel caused by the impact of the current COVID-19 epidemic, we only measured clay loam and loess for the secondary calibration of the sensor. To make the sensor useable with a variety of different soil types, future research should collect multiple soil types to build secondary calibration model libraries. In order to minimize the effect of compaction, we controlled the compaction of the calibration sample at a uniform level during the calibration process. The calibration of the EEMFI sensor was performed so that, after the calibration of the EEMFI sensor was completed, the soil volumetric water content value would be measured in actual use. This was then converted into the soil volumetric water content value at the same compaction level so that we could easily perform a comparative analysis of changes in soil moisture. In a subsequent study, we plan to investigate a device that can obtain the soil compaction degree at the same time and build a compensation model to further avoid the effect of soil compaction degree in the actual measurement.

At present, the EEMFI sensor we designed can only achieve the online continuous measurement of soil moisture in a small area; for the measurement of soil moisture in a larger area, our current research idea is to collect the microenvironmental meteorological parameters of the corresponding area, combined with satellite remote sensing images, and attempt to use artificial intelligence algorithms for data training in addition to using EEMFI. The results obtained from sensor measurements would be used as true values for model validation to examine whether a model algorithm can be established to achieve soil moisture measurements in larger areas. Meanwhile, we have not yet tested this method in a paddy field. In secondary calibration, when the moisture in the soil reaches saturation, the sensor edge electromagnetic field detection circuit output voltage value also no longer increases; therefore, for application in paddy fields, it is only suitable for measuring the soil moisture level when it is less than saturation; when the moisture in the soil is greater than saturation, it cannot be used.

## 4. Conclusions

In this study, we designed a low-cost sensor for soil water content detection based on the method of impedance detection using EEFMI and proposed a normalized calibration method to eliminate the errors caused by the measurement sensor’s characteristics and improve the consistency of the output. Comparing the traditional calibration with the normalization calibration proved that the proposed calibration method can effectively reduce calibration times, improve calibration, reduce the number of calibrations, and improve calibration efficiency. The proposed EEFMI water measurement probe has better versatility, easier maintenance and replacement, and more use in practical applications than previous probes. The normalized and quadratic calibration models were established through calibration experiments, and the fitted coefficients of determination exceeded 0.98.

The experimental results showed that the EEMFI sensor’s measurements had a water content range of 0% to saturation, measurement accuracy of ±2%, extreme value distribution of 1.09%, resolution of 0.05%, and a delay of 3.9 s. This proved that the EEMFI sensor has good static and dynamic characteristics to achieve actual soil water content sensor requirements. By analyzing the sensitive area of the EEMFI sensor measurement probe, we found that the effective measurement diameter of the EEMFI sensor was 10 cm. By comparing the measurement results with the internationally recognized TDR water content sensor and the drying method, the linear fit coefficient of determination between the EEMFI and TDR sensor measurement results was greater than 0.99, and their accuracy was comparable. The maximum absolute error between the EEMFI sensor measurement results and the drying method was 1.74%, which meets the actual requirements of soil water content measurement. The field experiment results showed that the EEMFI sensor can accurately respond to changes in soil water content, indicating that the sensor is reliable. The EEMFI sensor designed in this study can provide an effective and low-cost (USD 100) method for the rapid measurement of soil water content in agriculture, forestry, and ecological environments. The method proposed in this paper can be combined with other parameter estimation algorithms [55,56,57] and microenvironmental parameters [58] to study soil moisture prediction problems at different time scales [59,60,61,62] and can be applied to other engineering application systems [63,64,65]. In addition, it can be combined with IoT technology to deploy multiple sensors in different time-domain spaces for multi-data variable collection [66] and analysis to further analyze the spatiotemporal characteristics and cause–effect relationships among variables [67].

## Figures and Tables

**Table 1:** Price breakdown of components and materials for the EEMFI sensor.

Serial Number	Components and Materials	Price (USD)	Serial Number	Components and Materials	Price ($)
1	Bimetal ring probe	10	7	MAX485ESA	3
2	STM32103C8T6	4	8	Power Control Module	7
3	STM8S103F2P6TR	1	9	Clock Control Module	6
4	CS4344	0.5	10	PCB fabrication	15
5	AD8226ARZ	3.5	11	3D Printing	10
6	Edge electromagnetic field detection circuit	30	12	Others (resistors, capacitors, inductors, cable wires)	10

**Table 2:** Prices of some commonly used soil water sensors (data from https://www.instrument.com.cn/demand/, accessed on 15 January 2021).

Serial Number	Name	Production Location	Brands	Price ($)
1	TDR310W	America	Acclima	415
2	SDI-12	America	Acclima	445
3	Soil-5MTE	China	BolunQiXiang	370
4	sm10	America	Spectrum Technologies	148
5	TRIME-PICO-IPH	German	IMKO	7413
6	FT-W485	China	FengTu	110
7	PICO-BT	German	IMKO	2965
8	HD2	German	IMKO	1800

**Table 3:** Dielectric constants of organic solvents.

Solution	C_2_H_6_O: H_2_O					CH_3_COOH: H_2_O				
Volume ratio	0:1	1:1	2:1	5:1	1:0	4:1	6:1	10:1	20:1	1:0
Dielectric constant	80.00	52.25	43.00	33.75	24.50	20.92	16.20	12.86	9.67	6.15

**Table 4:** Fitting equations for the output voltage of the water content measurement probe and soil water content.

SOIL TYPE	Fitting Equation	$R^{2}$	K	B
Clay loam soil	θ=43.92U+8.24	0.98	43.92	8.24
Loess soil	θ=48.84U+6.24	0.99	48.84	6.22

**Table 5:** EEMFI sensor measurement accuracy.

Soil Type	Soil Sample Water Content (%)	EEMFI Sensor Measurement Results (%)	Measurement Accuracy (%)
Clay loam soil	13.72	13.40	0.32
	35.80	37.62	1.82
	41.82	40.18	1.64
Loess soil	17.88	16.47	1.41
	28.97	26.99	1.98
	43.27	44.39	1.12

**Table 6:** EEMFI sensor dynamic characteristics index.

Dynamic Characteristic	Overshoot (%)	Transition Time (s)	Delay Time (s)	Rise Time (s)	Peak Time (s)	Number of Oscillations
Results	0.87	2.9	3.9	1.8	6.0	0

**Table 7:** Comparison of measurement results between EEMFI sensor and drying method.

Experimental Site Number	EEMFI Sensor Measurement Results (%)	Drying Method Measurement Results (%)	Absolute Error (%)
1	11.7	13.22	1.52
2	13.9	12.18	1.72
3	10.2	11.94	1.74
4	9.8	10.78	0.98
5	21	20.3	0.7
6	12.9	13.26	0.36
7	18.4	17.92	0.48

## Data Availability

The data that support the findings of this study are available from the corresponding author upon reasonable request.
